# Bacterial composition along the digestive tract of the Horned Screamer (*Anhima cornuta*), a tropical herbivorous bird

**DOI:** 10.7717/peerj.14805

**Published:** 2023-02-13

**Authors:** María Alexandra García-Amado, Carla A. Rudolf, Maria del Mar Fuentes-Fuentes, Nataliya Chorna, Leoncia Margarita Martínez, Filipa Godoy-Vitorino

**Affiliations:** 1 Laboratorio de Fisiología Gastrointestinal, Centro de Biofísica y Bioquímica, Instituto Venezolano de Investigaciones Científicas, Miranda, Venezuela; 2 Microbiology Department, University of Puerto Rico, School of Medicine, San Juan, Puerto Rico; 3 Biochemistry Department, University of Puerto Rico School of Medicine, San Juan, PR, Puerto Rico; 4 Colección Ornitológica Phelps, Caracas, Venezuela

**Keywords:** Tropical bird, Microbiota, Herbivore, Cellulose degradation

## Abstract

**Background:**

The Horned Screamer *(Anhima cornuta)* is an herbivorous bird that inhabits wetlands of the South American tropical region. We hypothesize that due to its herbivorous niche, its digestive tract compartments may have bacteria specialized in fermenting complex plant carbohydrates. To test this hypothesis, we compared the bacterial communities along the gastrointestinal tract (GIT) of a Horned Screamer captured in Venezuela.

**Methods:**

Samples were taken from tissues and content of the proventriculus and the small intestine (considered for this study as upper GIT), and the large intestine and cecum (lower GIT). The bacterial community was characterized by sequencing the V4 region of the *16S rRNA* gene. Bioinformatic analysis was performed using QIIME, QIITA and Microbiome Analyst. The association between microbial taxonomy and function was analyzed using their Greengenes OTU IDs and a custom KEGG BRITE hierarchical tree and visualized with BURRITO.

**Results:**

The Screamer’s gastrointestinal microbiota was composed by seven phyla being Firmicutes and Bacteroidetes the most predominant. The dominant taxa in the upper GIT were *Helicobacter, Vibrio, Enterobacter, Acinetobacter* and *Staphylococcus*. The dominant taxa in the lower GIT were *Oribacterium, Blautia, Roseburia, Ruminococcus, Desulfovibrio, Intestinimonas, Marvinbryantia* and *Parabacteroides*. Complete degradation of cellulose to the end-products acetate, propanoate, butanoate and acetoacetate was found in the upper and lower GIT without significant differences.

**Conclusion:**

Our study confirmed changes in bacterial community composition throughout the GIT of the Horned Screamer primarily associated with the production of metabolic end-products of carbohydrate digestion essential for the fermentation of the herbivorous diet.

## Introduction

For some herbivorous vertebrates, leaves are an abundant and accessible food; however, animals do not have enzymes to degrade the complex carbohydrates of plant cell walls. As a result, microorganisms of their digestive tract are responsible for degrading these components through fermentation in specialized chambers in the gastrointestinal tract (GIT) (Parra, 1978; Stevens & Hume, 1998).

Avian folivory is unusual, with less than 3% of species depending on leaves in their diets—as the bulky fermentation chambers conflict with the high energetic requirements of flight. For this reason, many folivorous birds are bigger and have reduced or no flight capacity (Morton, 1978). To avoid these problems, some birds, such as ducks and geese, use only the soluble content of leaves without fiber digestion, so they ingest large amounts which pass quickly throughout their digestive tracts (Buchsbaum, Wilson & Valiela, 1986; Dawson, Johns & Beal, 1989). However, other birds that depend mainly or exclusively on leaves, could have post gastric fermentation chambers in the large intestine or ceca, such as the Ostrich (*Struthio camelus*), Rhea (*Rhea americana*) and Emu (*Dromaius novaehollandiae*), while the Hoatzin is the only known bird that has a pregastric fermentation chamber in the crop (Domínguez-Bello et al., 1994; Grajal, 1995; Grajal & Parra, 1995; Godoy-Vitorino et al., 2008, 2012a, 2012b).

The microbiota of avian species has coevolved with its host and plays a vital role in food digestion, production of nutrients, protection against pathogens, and regulation of the immune system (Ley et al., 2008; Wu & Wu, 2012; Waite & Taylor, 2014, 2015; Bodawatta et al., 2022). Studies of wild bird microbiomes have recovered interest in recent years, showing that the GIT bacterial communities differ significantly from those of other vertebrates (Ley et al., 2008; Godoy-Vitorino et al., 2012b; Waite & Taylor, 2015; Bodawatta et al., 2022). However, studies about microbiota characterization in herbivores wild birds are limited to a few species like the Hoatzin (Godoy-Vitorino et al., 2012a), the Kakapo (Waite & Taylor, 2012), the Greater Sage-Grouse (Kohl et al., 2016), and some members of the Anatidae family (Yang, Deng & Cao, 2016; Hao et al., 2021).

The Horned Screamer (*Anhima cornuta*) is a member of Anhimidae family (Anseriformes order), with two more species: the Southern Screamer (*Chauna chavaria*) and the Northern Screamer (*Chauna torquata*). These are aquatic birds found only in South America, and their conservation status is Least Concern by the IUCN (Hilty, 2003; BirdLife International, 2022). Horned Screamers are distributed in tropical lowland freshwaters of Venezuela, the eastern llanos of Colombia, eastern Bolivia and south-central Brazil (Naranjo, 1986; Piland, 2022). Horned Screamers have many unusual anatomical features like a long “unicorn” quill on the top of the head, two sharp-pointed spurs on each wing, and the legs and unwebbed toes unusually large and strong; the middle toe is exceptionally long to walk easily over floating vegetation, despite their great weight (Hilty, 2003). Despite being considered without conservation risk, the Horned Screamer populations in Venezuela are low (601 observations) in comparison with those in Colombia (3,496 observations) or with other herbivore birds like the Hoatzin (1,052 observations in Venezuela) (https://ebird.org). Additionally, the Horned screamer is a territorial bird, with a home range of 10.7 ha. The individuals are found alone or in couples; they seldom can form groups, but these groups are rarely larger than six (Naranjo, 1986; Piland, 2022). The Horned Screamer eats a variety of small shrubs and buds, grazes on *Hydrangea* spp., *Eichornia crassipes*, *Polygonum hispidum*, *Paspalum dilatatum* and *Artemisa absinthium* and continuously digs along the water (Naranjo, 1986; Hilty, 2003; Piland, 2022). We hypothesized that due to its herbivorous diet, the compartments along its GIT must contain bacteria capable of fermenting complex carbohydrates of plants. To test this hypothesis, we aimed to characterize the digestive bacterial community and its potential functions along the gastrointestinal tract of the Horned Screamer.

## Materials and Methods

### Sample collections

An adult male Horned Screamer, 2.5 Kg in weight, 79.0 cm total length and 210.0 cm wingspan, was hunted in San Pablo de Urama wetlands, Carabobo state, Venezuela (approximate coordinates: 10°31′14.7″N, 68°23′7.4″W) under the hunting permit for scientific purposes provided by the Ministerio del Poder Popular para el Ecosocialismo y Aguas (MINEC No 1727) and with approval of the IVIC Animal Ethics Committee (COBIANIM Dir-0884/1517). In our study area in Venezuela, we only found two individuals; and the second flew once we caught it. We returned some days after and did not see any other individual. As explained in the introduction, the *Horned Screamer* populations in Venezuela are low and primarily found in couples, hence using only one individual.

In the field, the animal was ventrally incised from throat to vent, to expose the gastrointestinal tract (GIT). After being removed from the body cavity, the GIT was dissected. The different organs (proventriculus, gizzard, small intestine, large intestine and cecum) were kept frozen in liquid nitrogen until arrival to the IVIC laboratory where the tissues were stored at −80 °C until DNA extraction.

**Figure 1:**
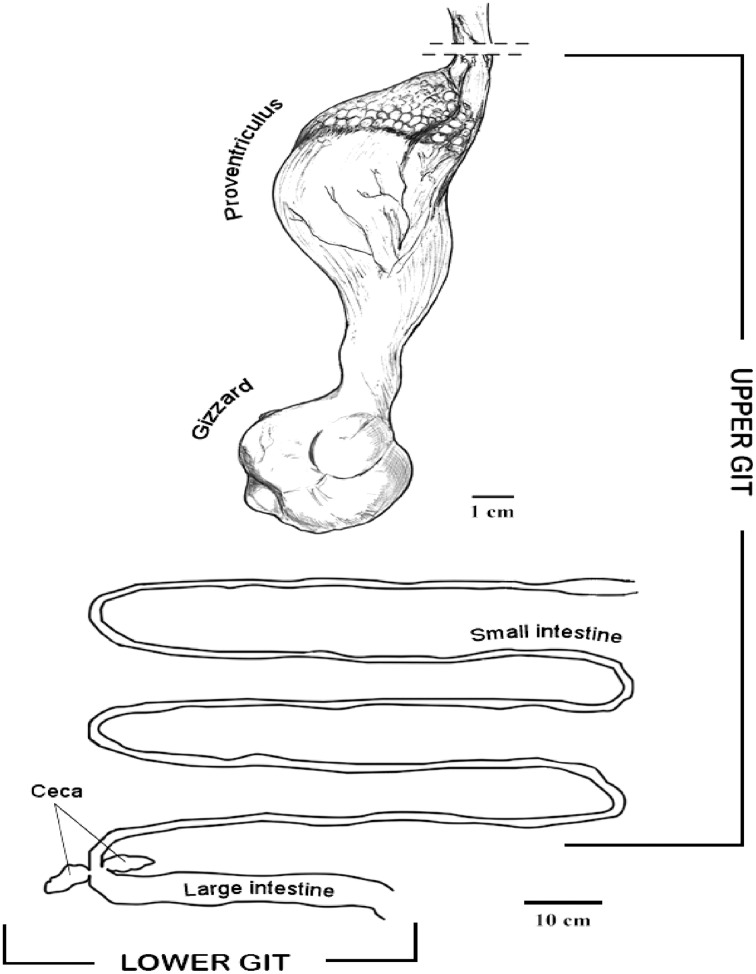
Diagram of the horned screamer gastrointestinal tract (GIT) sections organized as upper GI and lower GI.

In the IVIC lab, the GIT was defrosted and samples (200 mg) from tissues and content were sterile placed in the PowerBead tubes to perform the DNA extraction using the PowerSoil DNA Isolation kit (MO BIO, Inc., Carlsbad, CA, USA). The digestive tract was dissected, and samples from tissues and GIT content were taken from different organs and sections, including proventriculus, gizzard, small intestine, large intestine and cecum. The proventriculus and the small intestine were considered for this study as upper GIT, and the large intestine and cecum as lower GIT for analyses purposes (Fig. 1).

### Sequence data processing and statistical analyses

DNA was extracted from these samples using the PowerSoil DNA Isolation kit (MO BIO, Inc., Carlsbad, CA, USA). DNA from the GIT content samples were normalized to 4 nM during 16S library prep and the hypervariable region V4 of the 16S ribosomal RNA gene (~291 bp) was amplified using the universal bacterial primers: 515F (5′GTGCCAGCMGCCGCGGTAA3′) and 806R (5′GGACTACHVGGGTWTCTAAT3′) in the Earth Microbiome Project (http://www.earthmicrobiome.org/emp-standard-protocols/16s/) (Caporaso et al., 2012) using previously reported conditions in projects from our group (Rodriguez-Barreras, Tosado-Rodriguez & Godoy-Vitorino, 2021; Ruiz Barrionuevo et al., 2022; Abarca et al., 2018). We used the Illumina MiSeq Reagent kit 2 × 250 bp to sequence the 16S amplicons. The 16S-rRNA reads were deposited in QIITA (Gonzalez et al., 2018) Bio project ID 12669, and the raw sequences are available in the European Nucleotide Archive ENA ID ERP135861.

Raw read pre-processing of the demultiplexed files in QIITA were processed with a Phred offset of 33, and default parameters. Forward reads were trimmed to 250 bp and a closed reference approach was selected for OTU picking using the SILVA reference database for taxonomy assignment with a minimum similarity threshold of 97% (Pruesse et al., 2007). The species table file in biom format was downloaded for downstream analyses using a locally run version of QIIME (Caporaso et al., 2010). Singletons and reads matching chloroplasts or mitochondria were removed from downstream analyses. Data were collected as previously described (Rodriguez-Barreras, Tosado-Rodriguez & Godoy-Vitorino, 2021; Ruiz Barrionuevo et al., 2022). Data analysis was done using a rarefaction level of 1,000 sequences per sample considering the gut sections: proventriculus *n* = 3, small intestine *n* = 1, large intestine *n* = 4 and cecum *n* = 2. Due to sequence quality and rarefaction, gizzard samples were removed and only 10 samples were analyzed. Data analyses was done by grouping samples into two main groups, upper GIT (proventriculus and small intestine *n* = 4) and lower GIT (large intestine and cecum *n* = 6). Community level analyses (beta diversity) were done by computing the pairwise Bray-Curtis distances between samples and plotted as 2D NMDS plot in microbiomeAnalyst (Dhariwal et al., 2017). Statistical significance between sample groups was assessed using the PERMANOVA test (Anderson, 2001).

Alpha diversity measures were estimated including richness (Observed, Chao 1), ACE Abundance-based coverage estimator (non-parametric) and Fisher diversity were plotted as boxplots and nonparametric statistical t-tests with Monte Carlo permutations were calculated. Barplots and heatmap revealing phyla and genus taxa were computed using the same parameters with MicrobiomeAnalyst (https://www.microbiomeanalyst.ca/). Linear Discriminant Analysis Effect Size (LEfSe) was done to identify significant features (*p*-value cutoff: 0.05; Log LDA Score: 2.0) discriminating lower and upper gastrointestinal tract samples (Segata et al., 2011). A supplementary file details the analyses (Supplemental Document).

### Taxon-function analyses

Taxon-function attributions in the bird’s digestive and intestinal systems related to cellulose and hemicellulose fermentations were analyzed by the BURRITO framework (McNally et al., 2018). *16S rRNA* OTU data were separated into two microbiome datasets: Upper GIT and Lower GIT. Attribution calculations were based on the original PICRUSt and a genomic content table characterized using the Greengenes OTU IDs. The association between taxonomy and function in both microbiome datasets (lower and upper GIT) were evaluated using their Greengenes OTU IDs and a custom KEGG BRITE hierarchical tree, which was composed using KO attributed to cellulose and hemicellulose metabolism, including starch and sucrose metabolism, galactose metabolism, glycolysis, pentose phosphate pathway, pyruvate, butanoate and propanoate metabolism. The summary levels of functional attributions for each taxon to each metabolic pathway involved in the fermentation of plant carbohydrates and average shares of each function attributed to each taxon were presented as a heatmap constructed using GraphPad Prism version 9 (GraphPad Software, San Diego, CA, USA). Metabolic network of the fermentation of plant carbohydrates were composed using KEGG BRITE hierarchical tree (Kanehisa & Goto, 2000) and identified KO functional data attributed to the fermentation of plant carbohydrates. The network was constructed using Cytoscape (Shannon et al., 2003).

## Results

### Bacterial composition between upper and lower GIT

A total of 123,022 good quality *16S rRNA* gene reads were used for downstream analyses from the 10 gut samples of the Horned Screamer. Data analyses were done using a rarefaction level of 1,000 reads.

**Figure 2:**
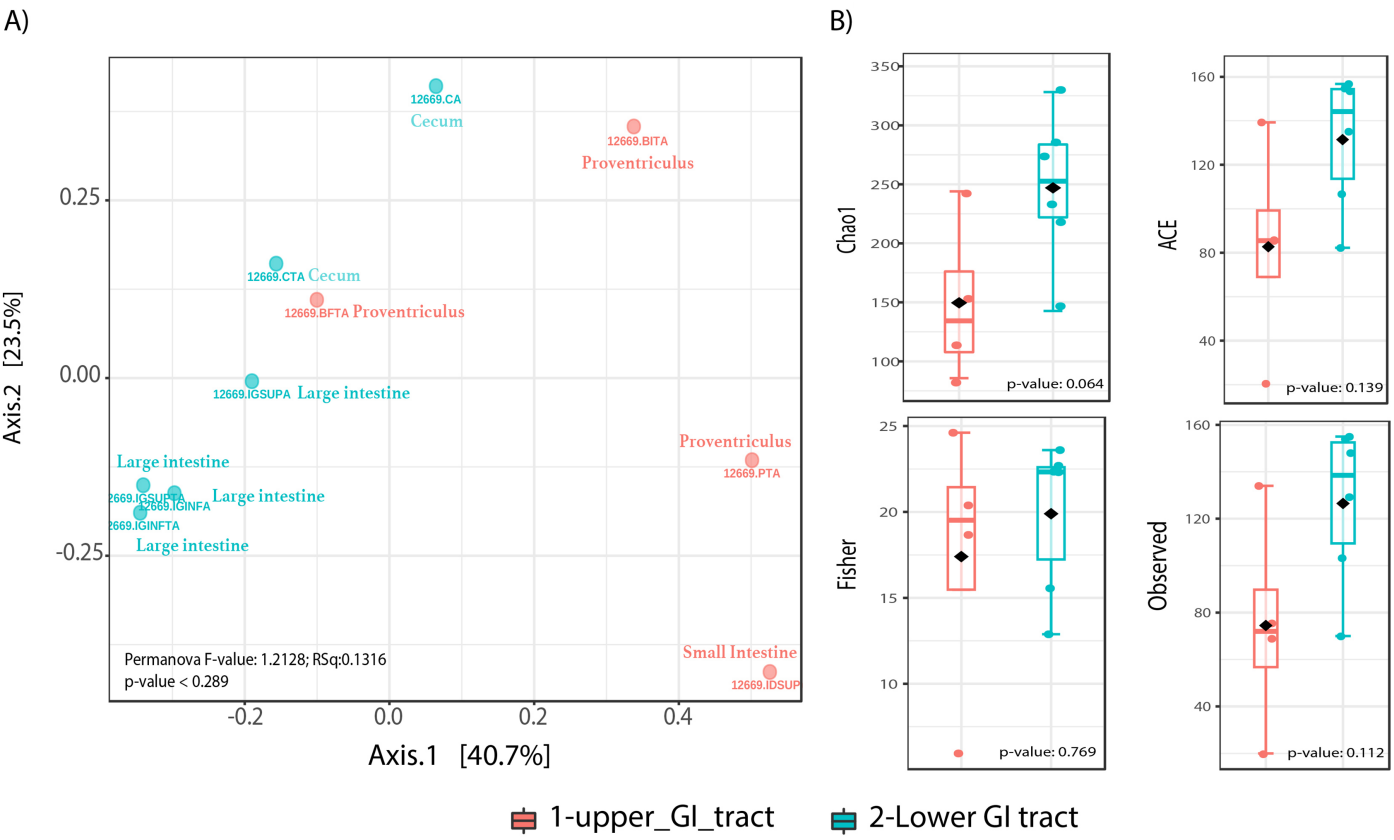
Microbiota community diversity comparing upper GI tract samples (crop, proventriculus and small intestine, *n* = 4) and lower GI tract samples (large intestine and cecum *n* = 6). (A) A beta diversity NMDS plot; (B) alpha-diversity plots as boxplots (Chao 1, ACE, Observed and Fisher Index).

Beta diversity analyses did not show significant differences in community structure or composition (Permanova *p*-value = 0.289), however the NMDS plot shows a distinction of upper GIT samples to the right of the plot x-axis, while most lower GIT samples converge to the left of the NMDS plot (Fig. 2A). Alpha diversity analyses among sites, showed nearly significant differences among GIT sites (Fig. 2B). The lower GIT samples had nearly significant higher alpha diversity (KW *p*-value: 0.064).

**Figure 3:**
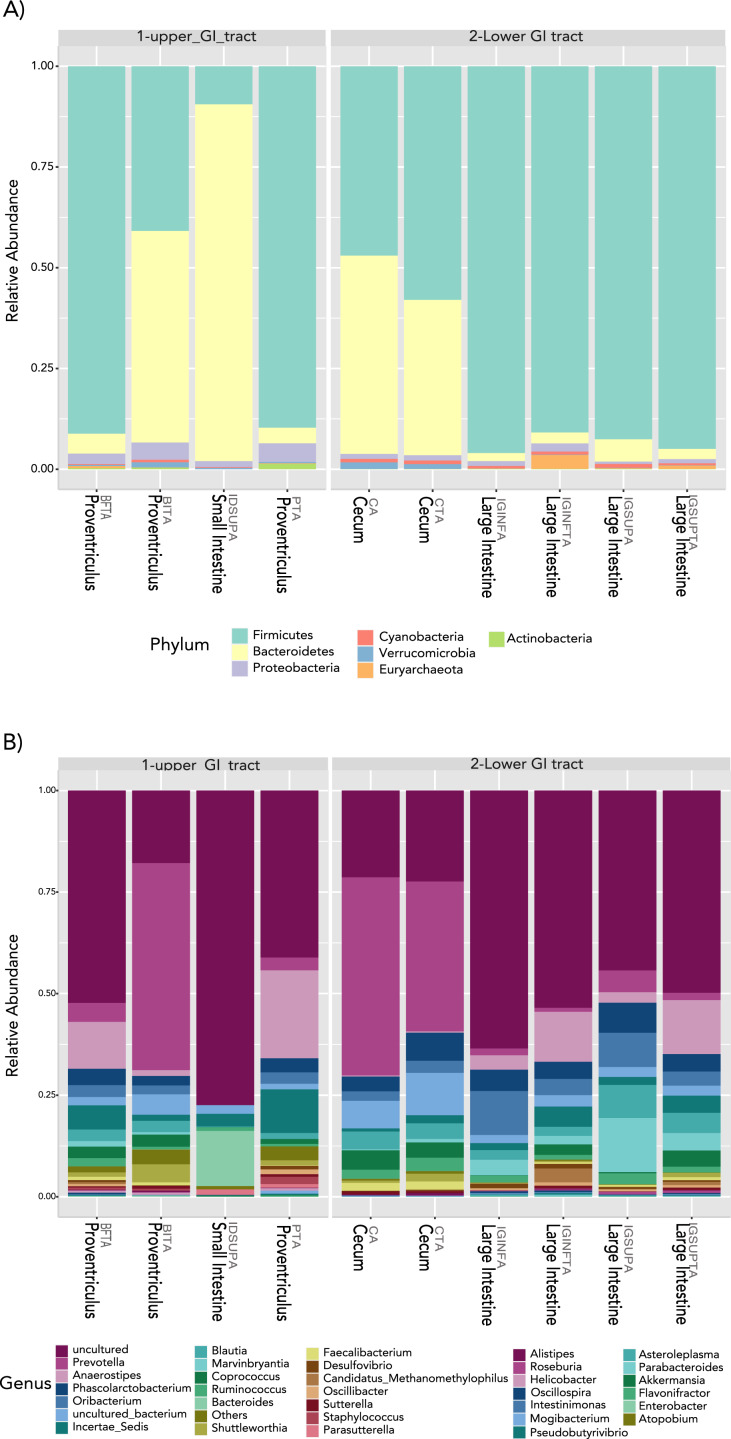
Taxonomy profiles at the bacterial communities of the *Anhima cornuta gut*. Taxonomy profiles are shown at the phyla (A) and genus levels (B).

**Figure 4:**
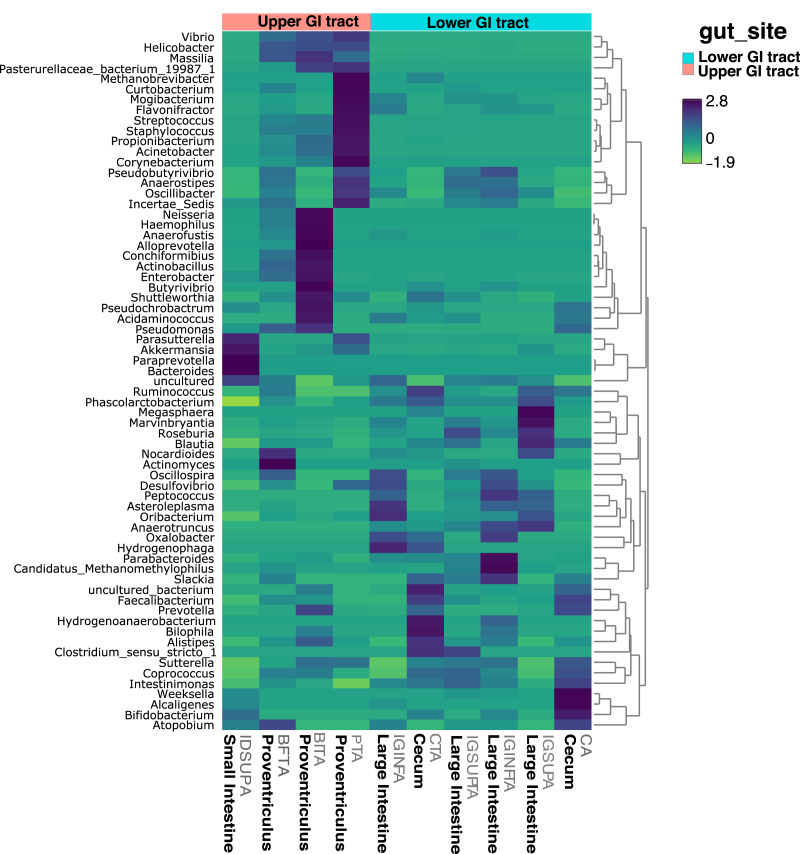
Heatmap showing the relative abundance of genus-level taxa according to lower and upper GIT.

In terms of composition, we found seven different phyla in the Screamer’s GIT. The most abundant phyla were Firmicutes and Bacteroidetes. Other phyla like Proteobacteria, Cyanobacteria, Euryarchaeota, Verrucomicrobia and Actinobacteria were found in lower abundance (Fig. 3A). At a genus level, a wider range of bacteria was found with uncultured species predominant. Some of the genera include *Prevotella*, *Anaerostipes, or Bacteroides* (Figs. 3B and 4).

**Figure 5:**
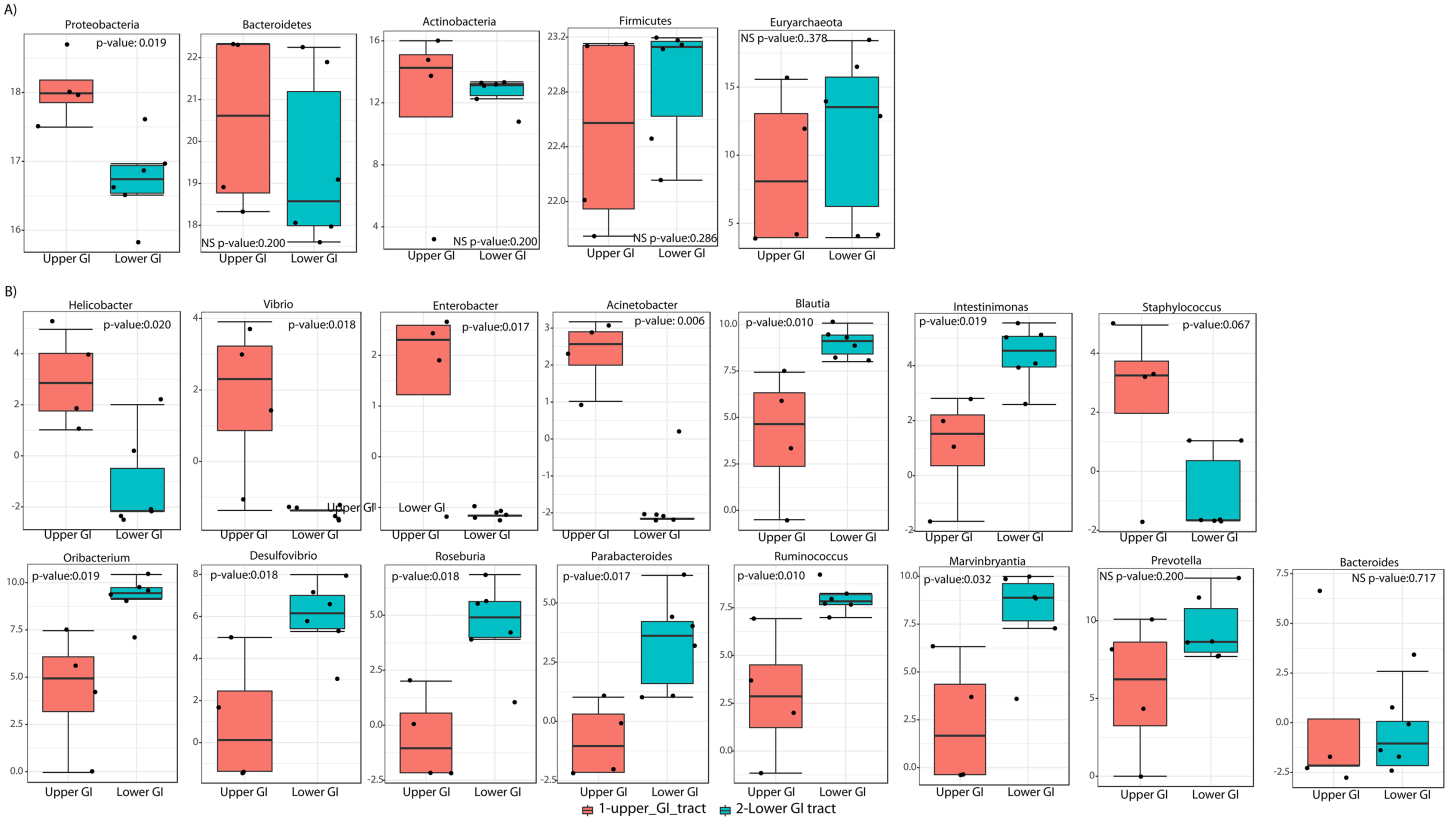
Linear discriminant analysis effect size (LEFsE) of bacterial communities. LEfSe box plot showing phyla-level differences between lower and upper GIT (A) and differences at the genus-level (B). Significantly different taxa are identified with *p*-values < 0.05 and an asterisk close to the taxa name.

LEfSe analyses determined that at the phyla level, Proteobacteria most likely to explain differences between upper and lower GIT (*p*-value = 0.019). Bacteroidetes and Actinobacteria were more dominant in the upper GIT although there were no significant differences (Fig. 5A). At a genus level, *Helicobacter, Vibrio, Enterobacter* and *Acinetobacter* (from the Proteobacteria phylum), had higher significant abundance in the upper GIT (Fig. 5B). *Blautia*, *Oribacterium*, *Roseburia*, *Ruminococcus*, *Intestinimonas*, *Marvinbryantia* (Firmicutes), *Desulfovibrio* (Proteobacteria) and *Parabacteroides* (Bacteroidetes) are more dominant in the lower GIT (Fig. 5B).

### Taxon-function attributions to metabolic pathways involved in the fermentation of plant carbohydrates

**Figure 6:**
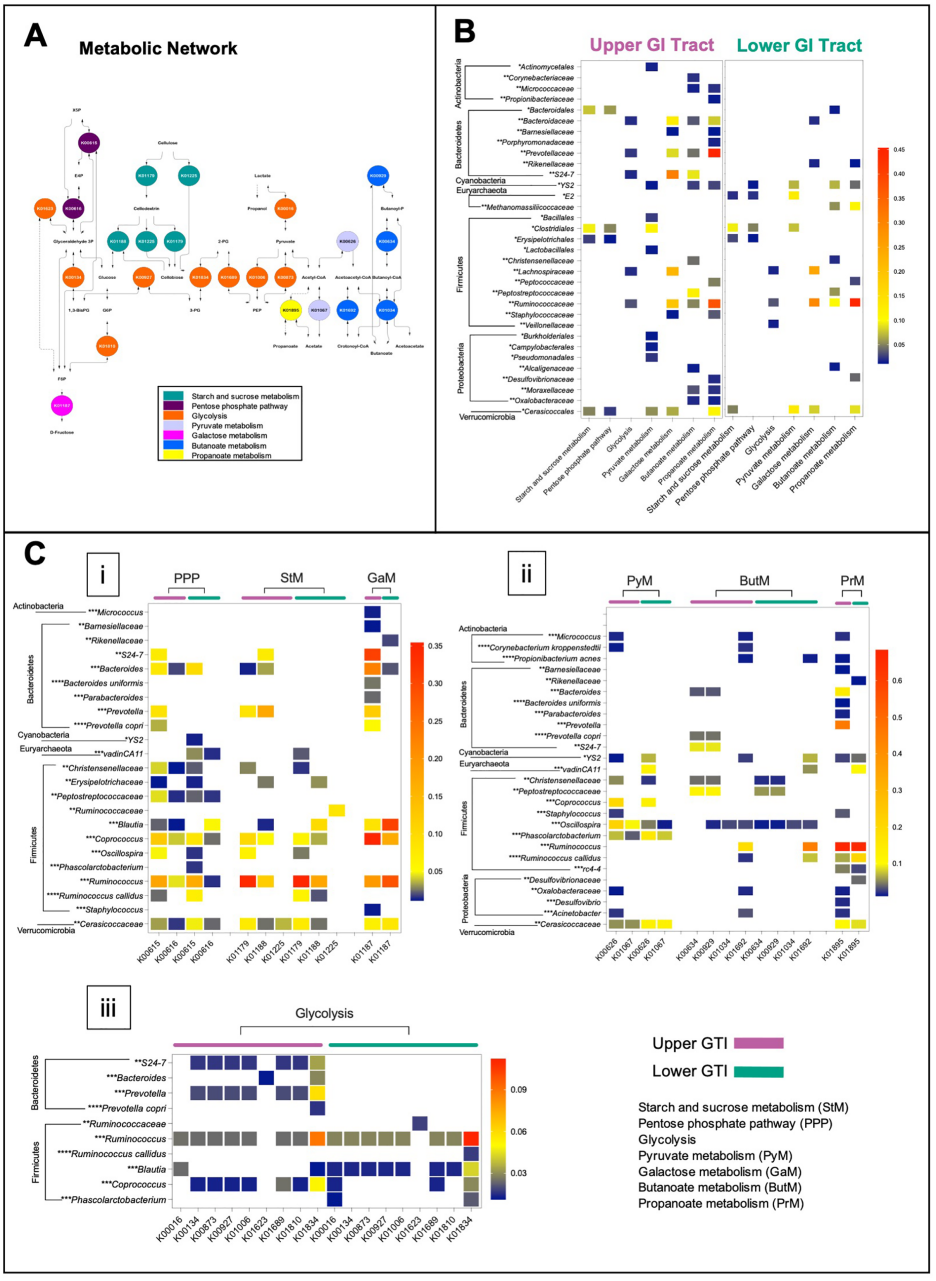
The functional capacity of the upper and lower GIT microbiomes in the *Anhima cornuta*. The functional capacity of the upper and lower GIT microbiomes in the fermentation of plant carbohydrates evaluated *via* the BURRITO framework. (A) Metabolic network of the fermentation of plant carbohydrates composed using KEGG BRITE hierarchical tree and identified KO functional data attributed to the fermentation of plant carbohydrates. (B) The heatmap shows the summary shares of functional attributions for each taxon to each metabolic pathway involved in the fermentation of plant carbohydrates. (C) The heatmap shows the average share of each function attributed to each taxon in each metabolic pathway. Red indicates the highest average share and blue—the lowest. Taxon-function attribution shares were selected based on cut-off <0.01. Abbreviations: PEP, Phosphoenolpyruvate; 3-PG, 3-Phosphoglycerate; 1,3-BisPG, 1,3-Bisphospho-D-glycerate; 2-PG, 2-Phospho-D-glycerate; G6P, Glucose 6-phosphate; F6P, Fructose 6-phosphate; E4P, Erythrose 4-phosphate; X5P, Xylulose 5-phosphate. Each taxon was grouped to correspondent phylum, and taxonomy levels were labeled with asterisks: *—order, **—family, ***—genus, and ****—species.

The microbiome functional capacity in the fermentation of plant carbohydrates was evaluated in the upper and lower GIT *via* the BURRITO framework (McNally et al., 2018). We identified 22 functions associated with starch and sucrose metabolism, galactose metabolism, glycolysis, pentose phosphate pathway, pyruvate, butanoate, and propanoate metabolism (Fig. 6A), and 28 species contributors. In both regions, *Firmicutes* was found as the main drivers of the activity of metabolic enzymes involved in the fermentation of plant carbohydrates. *Bacteroidetes* and *Proteobacteria* mainly contributed to many metabolic reactions in the upper GIT (Figs. 6B and 6C).

In the lower GIT, only two genera of *Bacteroidetes*, such as *Bacteroides*, contributed to galactose metabolism and *Rikenellaceae—*to galactose metabolism and butanoate metabolism. On the other hand, among *Proteobacteria* identified in the lower GIT, *Desulfovibrionaceae* contributed to butanoate metabolism and propanoate metabolism correspondingly (Figs. 6B and 6C). *Actinobacteria*, including *Corynebacterium*, *Micrococcus*, and *Propionibacterium*, contributed to pyruvate metabolism, galactose metabolism, butanoate metabolism, and propanoate metabolism only in the upper GIT (Figs. 6B and 6C). In contrast, *Methanomassiliicoccaceae_vadinCA11* (*Euryarchaeota*) contributed with many metabolic reactions only in the lower GIT (Figs. 6B and 6C). *Cerasicoccaceae* (*Verrucomicrobia*) *and Cyanobacteria_YS2* also contributed to many metabolic reactions in both anatomic regions (Figs. 6B and 6C).

### Metabolic functions associated with gut microbiome on the fermentation of plant carbohydrates

It is recognized that the central metabolic hub in the process of fermentation of carbohydrates is glycolysis. Cellulose (CL), a monomeric polymer of glucose, was converted into cellodextrin and cellobiose by endoglucanase (K01179) and cellulose 1,4-beta-cellobiosidase (K01225), followed by breaking cellobiose into glucose by beta-glucosidase (K01188) in all regions (Fig. 6C, i). Identification of possible taxonomic drivers of hemicellulose (hCL) fermentation, a more complex heterogeneous polysaccharide, did not produce any conclusive results with some exceptions, such as the microbial involvement in the conversion of galactose and xylose in both regions. In particular, the digestion of galactose to glucose was associated with alpha-glucosidase activities (K01187), while the digestion of xylose to fructose-6-phosphate, erythrose-6-phosphate, and xylulose-5-phosphate through the pentose phosphate pathway was concomitant with the activity of transketolase (K00615) (Fig. 6C, i).

We also analyzed the functional capacity of microbiota in yielding end-products acetate, propanoate, butanoate, and acetoacetate (Fig. 6C, ii). Thus, propanoate produced from propanoyl-CoA by acetyl-CoA synthetase (K01895) was predicted for both regions and associated with *Firmicutes*, *Bacteroidetes*, *Proteobacteria*, *Cyanobacteria*, *Euryarchaeota*, and *Verrucomicrobia*. In the upper GIT, *Prevotella* and *Ruminococcus*, and in the lower GIT *Ruminococcus* were the most robust contributors for this metabolic reaction (Fig. 6C, ii). The contribution of *Actinobacteria* for propanoate production was predicted only in the upper GIT (Fig. 6C, ii). Contribution to butanoate and acetoacetate production in the upper GIT was associated with *Firmicutes*, *Bacteroidetes*, *Proteobacteria*, and *Verrucomicrobia*, where the strongest contributors were *Ruminococcus*, *Peptostreptococcaceae*, and *Bacteroidales_S24-7* (Fig. 6C, ii). In the lower GIT, butanoate production was concomitant with *Firmicutes*, *Proteobacteria*, *Cyanobacteria* and *Euryarchaeota*, and *Ruminococcus* was the most robust contributor to this metabolic reaction (Fig. 6C, ii). Next, we identified that the production of acetate by acetyl-CoA hydrolase (K01067) in both queried regions was associated with *Firmicutes*, *Cyanobacteria*, and *Verrucomicrobia*. In addition, *Actinobacteria* and *Proteobacteria* also contributed to this metabolic reaction in the upper GIT. Examining the shares in acetate production revealed that *Coprococcus* was the strongest contributor in both regions, while *Oscillospira* in the upper GIT and *Phascolarctobacterium*, *Cerasicoccaceae Methanomassiliicoccaceae_vadinCA11* in the lower GIT (Fig. 6C, ii). Conversion of fructose-6-phosphate to glyceraldehyde-3-phosphate and 3-phosphoglycerate were associated with the activity of transaldolase (K00616) and glyceraldehyde 3-phosphate dehydrogenase (K00134) and fructose-bisphosphate aldolase (K01623) (Figs. 6C, i and iii). Several glycolytic enzymes such as L-lactate dehydrogenase (K00016), 2,3-bisphosphoglycerate-dependent phosphoglycerate mutase (K01834), enolase (K01689), glucose-6-phosphate isomerase (K01810), pyruvate kinase (K00873), and phosphoglycerate kinase (K00927) were predicted to be associated with either Firmicutes or Bacteroidetes (Fig. 6C, iii).

## Discussion

The Horned Screamer microbiota varied among each GIT site and was primarily composed by Firmicutes and Bacteroidetes phyla. The shared core microbiota of wild birds is dominated by members of four major phyla: Firmicutes, Proteobacteria, Bacteroidetes and Actinobacteria (Grond et al., 2018). The Horned Screamer’s microbiota is more similar to the reported in other herbivore mammals like cows (Terry et al., 2019), goats (Wang et al., 2019), capybaras (Cabral et al., 2022) and lemurs (Greene et al., 2019), herbivore reptiles like iguanas (Hong et al., 2011) and herbivore birds like Hoatzin (Godoy-Vitorino et al., 2012a), Sage-Grouse (Kohl et al., 2016) and White Pekin Duck (Hao et al., 2021).

We observed an abundance of Firmicutes across the GIT—which play a key role in fiber degradation (Flint et al., 2012); however, these predominated in the proventriculus (upper GIT) and large intestine (lower GIT). These bacteria can produce short-chain fatty acids as a result of the fermentation. The host gut wall can absorb these fatty acids as an energy source (Grond et al., 2018). Our data shows the activity of metabolic enzymes from Firmicutes taxa in the fermentation of plant carbohydrates.

Bacteroidetes were more abundant in the small intestine (upper GIT), with functional capacity associated with propanoate, butanoate and acetoacetate production, while in the lower GIT (mostly cecum), associated with galactose and butanoate metabolism. Bacteroidetes is a predominant phylum also in Capybaras, and it is associated to hemicellulose and pectin degradation, polysaccharides found in gramineous and aquatic plants (Cabral et al., 2022). In Venezuela, Horned Screamers live in a similar habitat than Capybaras and probably consume a similar diet.

*Bacteroides* were dominant in the upper GIT (mostly small intestine), a genus also found to be abundant in another waterbird, the Mallard (*Anas platyrhynchos*) (Boukerb et al., 2021). *Bacteroides* spp. are characterized by having a strong ability to degrade protein and polysaccharides. They play a specific role in the breakdown of cellulose and other plant materials that can contain rich nutrients for the host (Grond et al., 2018). They also have a metabolism mainly based on the degradation of dietary and mucus glycoproteins which are key in the immune system regulation (Grond, Guilani & Hird, 2020) and in the plant secondary metabolites detoxification in other herbivores birds like Rock Ptarmigans (Salgado-Flores et al., 2019) and Sage Grouse (Kohl et al., 2016). In some plants consumed by the Horned Screamer, such as *Hydrangea* spp. and *Eichornia crassipes*, the presence of toxic secondary compounds have been reported as a defense against herbivorous insects (Ujihara, Shinozaki & Kato, 1995; Lalitha, Sripathi & Jayanthi, 2012) and *Bacteroides* likely detoxifies these plant secondary compounds.

In the proventriculus (upper GIT) and the cecum (lower GIT) sections, *Prevotella* was the most predominant bacteria. *Prevotella* is common in other herbivore birds like Rock Ptarmigans (Salgado-Flores et al., 2019) and Sage Grouse (Kohl et al., 2016). In the upper GIT, *Prevotella* was mostly associated with the metabolism of starch, sucrose, galactose, propanoate and butanoate. As reported in the rumen—where most *Prevotella* species use starch, xylan and pectins—this seems to be a common metabolic route to release nutrients from plant cell wall breakdown (Flint & Duncan, 2014).

Wild waterbirds are known as reservoirs of enteric bacterial pathogens such as *Campylobacter* spp., *Helicobacter* spp. *Clostridium* spp. and *Salmonella* spp. (Waldenström et al., 2007; Ryu et al., 2014; Smith, Snyder & Owen, 2020; Boukerb et al., 2021). In the Horned Screamer, potential human pathogens like *Helicobacter*, *Vibrio*, *Enterobacter* and *Acinetobacter* were also found. However other techniques like culture-based isolation, screening antibiotic resistance and virulence genes are necessary to define the bacterial species and evaluate their pathogenicity.

## Conclusions

The Horned Screamer microbiota changes throughout the GIT and is primarily composed by Firmicutes and Bacteroidetes phyla, similar to that reported in others herbivore vertebrates. The taxon-function analyses confirm that bacterial community in the Horned Screamer GIT is associated to complex carbohydrate fermentation of its herbivorous diet.

## Supplemental Information

10.7717/peerj.14805/supp-1 Supplemental Information 1 Data analyses pipeline from QIITA to Microbiome Analyst. Detailed methodological processes

## Additional Information and Declarations

### Field Study Permissions

The following information was supplied relating to field study approvals (*i.e*., approving body and any reference numbers): Field experiments were approved by Caza científica #1727 provided by the Ministerio del Poder Popular para el Ecosocialismo y Aguas (MINEC No 1727).

### Data Availability

The following information was supplied regarding data availability: The sequenced reads are available in the European Nucleotide Archive (ENA): ERP135861.
