# Supplementary material for: Bacterial composition along the digestive tract of the Horned Screamer (Anhima cornuta), a tropical herbivorous bird
Source: PeerJ. 2023 Feb 13;11:e14805. doi: 10.7717/peerj.14805 (PMC9933741; doi:10.7717/peerj.14805)
Supplement: Supplemental Information 1 — Detailed methodological processes [file peerj-11-14805-s001.pdf]

Supplementary Information

“Bacterial composition along the digestive tract of the Horned Screamer (*Anhima cornuta*), a tropical herbivorous bird”

Data analyses pipeline from QIITA to Microbiome Analyst

QIITA project downloaded Biom corresponded to 250bp trimming, SILVA reference database (closed reference).

Removed singletons, chloroplasts and mitochondria: * otu_table-clean.biom

- **qiime tools import --type FeatureTable[Frequency] --input-path** 250screamer-77694_otu_table-clean.biom **--output-path** screamer-otu-table-clean.qza
- **time qiime diversity alpha --i-table** screamer-otu-table-clean.qza **--p-metric 'chao1' --o-alpha-diversity** screamer-alpha-diversity-chao1.qza
- **time qiime diversity alpha --i-table** screamer-otu-table-clean.qza **--p-metric 'shannon' --o-alpha-diversity** screamer-alpha-diversity-shannon.qza
- **qiime diversity alpha-group-significance --i-alpha-diversity** screamer-alpha-diversity-chao1.qza **--m-metadata-file** Metadata_screamer_qiime_real.txt **--o-visualization** screamer-chao1-group-significance.qzv
- **qiime diversity alpha-group-significance --i-alpha-diversity** screamer-alpha-diversity-shannon.qza **--m-metadata-file** Metadata_screamer_qiime_real.txt **--o-visualization** screamer-shannon-group-significance.qzv

Using MicrobiomeAnalyst https://www.microbiomeanalyst.ca/MicrobiomeAnalyst/
- **Marker Data Profiling**
- R studio (to create alpha and beta diversity plots) BIOM FORMAT
- Chose Biom file and metadata Taxonomy labels SILVA submit
  >Default parameters, used 1,000 read minimum rarefaction level.
  Alpha diversity boxplots, Taxonomic profiles, Heatmap and LEFSE, were all plotted using the same parameters in MicrobiomeAnalyst

RStudio (betadiversity plot NMDS)

```
> library(tools)
> library(phyloseq)
> library(ggplot2)
> library(stringr)
```

```
> library(plyr)
> library(knitr)
> library(rmarkdown)

> set.seed(100)

> setwd("~/Documents/Universidad/Investigación/Data Screamer/clean data/R studio ")

> abund_table<-read.csv("OTU_table_R_screamer.csv",sep = ",", header = TRUE,
row.names=1,check.names=FALSE)
> abund_table<-t(abund_table)

> meta_table<-read.csv ("Mapping_Data_Screamer.csv" ,sep = ",", header =
TRUE,row.names=1,check.names=FALSE)

> OTU_taxonomy<-read.csv("taxa_screamer_R.csv", sep = ",", header =
TRUE,row.names=1,check.names=FALSE)

> OTU = otu_table(as.matrix(abund_table), taxa_are_rows = FALSE)
>TAX = tax_table(as.matrix(OTU_taxonomy))
> SAM = sample_data(meta_table)

> physeq <- merge_phyloseq(phyloseq(OTU, TAX, SAM))
> sample_names(SAM)
> sample_names(OTU)

> physeq
> sample_data(physeq)
> color <- c("#b2182b","#4393c3","#f46d43","#66bd63")

#Beta diversity
> set.seed(100)
>theme_set(theme_bw())
>ord.res <- ordinate(physeq, distance = "bray", method = "NMDS", grouping_column = "gut_site_sort",
pvalue.cutoff = 0.05)

>#Samples by category- Gut Site Sort
>ord <- plot_ordination(physeq, ord.res, type = "samples", color="gut_site_sortA")

>ord + theme_bw() + theme(text = element_text(size = 16))

>ord + geom_point(size=6, alpha=1) +
  scale_color_manual(values = color) +
  theme(scale_fill_manual(values = color)) -> p2

>ggsave("gut_site_sort-beta-div.pdf", width = 6, height = 4, units = "in")
>library(Screamerbird)
>grid1 <- plot_grid(p1,p2)
>ggsave("screamer_Beta_Div.pdf", width = 20, height = 5, units = "in")

>library(vegan)
metadata <- as(sample_data(physeq), "data.frame")

>adonis(distance(physeq, method="bray") ~ gut_site_sortA,
      data = metadata)
```
